# Isolation, culture, characterization, and osteogenic differentiation of canine endometrial mesenchymal stem cell

**DOI:** 10.14202/vetworld.2017.1533-1541

**Published:** 2017-12-29

**Authors:** A. K. Sahoo, J. K. Das, S. Nayak

**Affiliations:** Department of Veterinary Surgery and Radiology, College of Veterinary Science and Animal Husbandry, OUAT, Bhubaneswar - 751 003, Odisha, India

**Keywords:** Alizarin Red-S staining, endometrium, mesenchymal stem cell, multipotent stem cells marker, osteogenic differentiation media

## Abstract

**Aim:**

In this study, the canine endometrium tissue is characterized for its stem cell properties such as adherence to tissue culture plate (plasticity), short population doubling time, serial clonal passaging, long-term culturing properties, stem cell marker expression, and multilineage differentiation potential.

**Materials and Methods:**

The present work describes a novel isolation protocol for obtaining mesenchymal stem cells from the uterine endometrium and is compared with cells derived from umbilical cord matrix as a positive control. These cells are clonogenic, can undergo several population doublings *in vitro*, and can be differentiated to the osteocytes in mature mesenchymal tissues when grown in osteogenic differentiation media as detected by Alizarin Red-S staining.

**Results:**

It is reported for the first time that the cells derived from the canine endometrium (e-multipotent stem cells [MSCs]) were able to differentiate into a heterologous cell type: Osteocytes, thus demonstrating the presence of MSCs. Thus, the endometrium may be told as a potential source of MSCs which can be used for various therapeutic purposes.

**Conclusion:**

The endometrium can be used as a potential source of MSCs, which can be used for various therapeutic purposes.

## Introduction

Stem cells are undifferentiated cells defined by their ability at the single-cell level to both self-renewal and differentiate to produce mature progeny cells which include both non-renewing progenitors and terminally differentiating cells. Those have been classified by their developmental potential as totipotent, pluripotent, multipotent, oligopotent, and ­unipotent [1]. Again, these are also classified as embryonic and adult stem cells as per its origin. The adult stem cells are very difficult to identify in tissues as they are rare, lack in distinguishing morphological features, and the specific adult stem cell markers are currently unavailable. They are also defined by functional properties such as substantial self-renewal, high proliferative potential, and ability to differentiate into one or more lineages [2-4]. The multipotent stem cells (MSCs) were first isolated and characterized from bone marrow [5] and later on from various tissues such as umbilical cord blood [6], adipose tissue [7], amniotic fluid [8], amniotic membrane [9], and peripheral blood [10]. The existence of endometrial mesenchymal stem cell (En-MSCs) population in different species has been demonstrated by different groups in heifer [11], human [12], murine [13], ovine [14], equine [15], and canine [16].

Recently, while looking into the numerous application of stem cells in regenerative medicine as a part of the therapeutic application, its isolation from a readily available source is need of the day which is ethically conducive, plenty available, low cost and still have the multipotent characteristic. The unlimited proliferative and developmental potential of endometrial stem cells offer considerable opportunity for applications in regenerative medicine and tissue engineering.

Till date very little information has been in the lit­erature about canine endometrium derived stem cells. Hence, in this study, attempts have been made to derive the adult stem cells from canine endometrium basing on by their functional properties such as adherence to tissue culture plate, (plasticity), short population doubling time, serial clonal passaging, long-term culturing properties, stem cell marker expression, and multilineage differentiation potential.

## Materials and Methods

### Ethical approval

Although the animal tissues were used in the research study for the harvesting of tissues, it did not involve any invasive or inhumane methodology. In fact, sacrificing live animals for harvesting the required tissues were not needed at all. The canine uterus and umbilical cord matrix used in the current research were collected during the routine spaying procedure and cesarean sectioning, for deliveries of the fetuses, respectively, in the Department of Veterinary Surgery and Radiology. The samples were also collected from routine spaying procedures being conducted at Veterinary Polyclinic Saheed Nagar, as Animal Birth Control Program, a flagship program of Fisheries and Animal Resources Development Department (F.A.R.D.), Government of Odisha. Due permission was obtained from the Directorate of Animal husbandry and Veterinary Services for obtaining tissue samples and further analysis in the study. Thus, discarded and disposed of materials collected from these centers were only used for isolating and characterizing stem cells from the tissue samples.

### Study design

Healthy mongrel breed dogs (n=8, B.wt=14-20 kg) were used for the study. Applicable institutional and governmental regulations concerning the ethical use of animals were followed during the research. The exteriorized discarded whole uteri were collected through ovariohysterectomy procedure following the standard operative protocol of Animal Welfare Board of India. In the spaying procedure, animals were premedicated intramuscularly with injection atropine (0.04 mg/kg), injection xylazine (1.0 mg/kg), and injection ketamine (5-10 mg/kg) and maintained on ketamine anesthesia intravenously with drip. A total of 12 numbers of samples (8 number of the uterus for scrapping and four number of umbilical cord from one caesarian section patient) were collected for experimental design. Maintenance of dogs was done in the kennel of ABC wing of Veterinary Polyclinic and was released at about 5-7 days after wounds have been healed. Three different transport media, i.e., liquid nitrogen (LN2), phosphate-buffered saline (PBS), and RNAlater^™^ stabilizing agents were used for collection and transfer of tissues to the laboratory.

The uterus which was snap frozen in liquid nitrogen was subjected to cryostat sectioning (Leica CM 1850, Germany) and mounted on a charged glass slide. The specimen on a glass slide was air dried, stained as per the protocol for hematoxylin and eosin (H and E) staining of frozen section, and was observed under light microscope (Leica, Germany). Tissues collected in 1× PBS were also subjected to paraffin block embedding and sectioning to undergo further H and E staining for histological views of three layers of the uterus (perimetrium, myometrium, and endometrium). This was done to confirm that the innermost layers which were scrapped to collect the tissue samples belong to the uterine endometrium containing prospective cells.

### Cell isolation and culture

**Figure-1:**
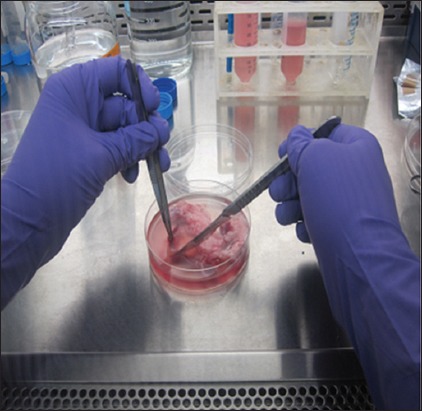
Scrapping of endometrium under sterile conditions.

**Figure-2:**
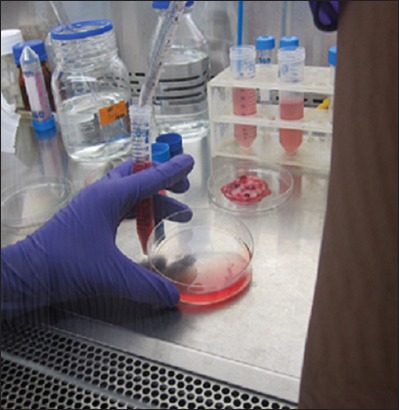
Trypsinization of sample.

For isolation and culture of cells, the collected uterine tissue samples were preserved in 1× PBS with 100 U/mL penicillin G and 100 µg/mL streptomycin and opened under laminar hood following strict aseptic measures. The sample was transferred to a 60 mm² Petridish (BD Falcon, USA) with sterile 1× PBS in it. Uterus was cut through and rinsed with sterile PBS several times, and the inner surface was scrapped to obtain thin tissue pieces (Figure-1). As per general histology, canine endometrium contains stratum basalis (base layer containing secreting glands) and stratum functionalis (contains side population cell and endometrial progenitor cell). Then, the scrapped endometrium was separated and the aliquot was transferred to a conical tube. The collected endometrial tissue was subjected to enzymatic and mechanical digestion in two steps as per Park *et al*. [9]:

Step 1: The collected endometrium was treated with trypsin - EDTA (0.25%) (trypsin (1×), 59429C, Safc, Bioscience USA) at 37°C. Then, it was incubated in a CO_2_ incubator (5% CO_2_, 0.4% O_2_) New Brunswick Incubator Galaxy 170R) chamber for 5 min. Then, the samples were taken out, washed with PBS, and the supernatant poured onto a separate 15 ml conical tube (Figure-2).

**Figure-3:**
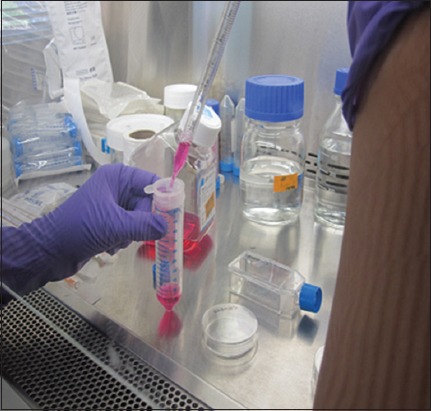
Passaging of trypsinized cells through cell strainer.

Step 2: The sample was centrifuged at 1500 rpm for 10 min at 37°C and the supernatant was discarded keeping the cell pellet in the test tube. Cells were passed through the cell strainer having 70 mm pore size (BD Bioscience, USA) and the flow through then transferred to a 15 ml conical tube and frequently mixed by pipetting with Easypet (Eppendorf) (Figure-3). After mechanical separation, enzymatic digestion, washing, straining, and purification, the cells were seeded into two different basal culture media in four different ways such as: (a) T_25_ polystyrene culture flask containing RPMI 1640+10% fetal bovine serum (FBS)+dexamethasone (maintained as reserve cells throughout the research work and preserved for future use), (b) 30 mm^2^ Petridish containing RPMI 1640+10% FBS+dexamethasone (used for RNA extraction on 14^th^ day), (c) T_25_ polystyrene cell culture flask containing Dulbecco’s modified Eagle’s medium (DMEM)+FBS 10% (used for induction media after 3 weeks of culturing), and (d) 30 mm^2^ Petridish containing DMEM+FBS10% (used as a control for induction media). All the above processing was done in cell culture laboratory under laminar hood maintaining the strict guidelines of sterilization and also avoiding all sorts of contamination. The prepared cell suspension was incubated in a humidified atmosphere with 5% CO_2_. The basal culture medium (RPMI 10%+dexamethasone and DMEM 10%) was changed 2 times a week and passaged, once the cells reached 80-90% confluence.

### RNA extraction and quantification

Total RNA was isolated according to the protocol outlined in the illustra™ RNAspin Mini RNA Isolation Kit (GE Healthcare, UK) following manufacturers guidelines. The RNA was eluted in 40 μl RNase-free H_2_O or storage solution (Riboreserve™ RNA storage solution, Amresco, Ohio) and incubated at room temperature for 10 min before being centrifuged at 11 000**×** *g* for 1 min. Eluted RNA was immediately placed on ice to prevent potential degradation. The eluted RNA was kept at −20°C for short-term storage and RNA present was quantified through spectrophotometer (Eppendorf Biophotometer plus, Germany). Spectrophotometry was done and the assessed RNA quality with nucleotide : protein ratio (260:280) was within acceptable boundaries of 1.8 and 2.1. 5 µL of freshly prepared uterine RNA were run on 1.5% agarose gel to check RNA band and quality. Band separation was first observed under UV transilluminator and then visualized using Molecular Imager^®^ Chemi Doc™ XRS+Imaging System, BIO-RAD with Image Lab™ software Version 3.0.

Extracted RNA was reversed transcribed using the protocol outlined in the High-capacity cDNA Reverse Transcription Kit (Applied Biosystem, USA) as in Table-1. 20 µL of reaction sample in polymerase chain reaction (PCR) tube was set in the thermal cycler with a setting of time and temperature as 25°C for 10 min, 37°C for 120 min, 85°C for 5 min, and 4°C for ∞ time. The newly formed cDNA (20 µL) was stored at −20°C.

**Table-1:** Preparation of cDNA Reverse transcription reaction.

cDNA reagent mixture	Amount	Concentration
10×RT buffer	2 µL	1×
25×dNTP mix	0.8 µL	4 mM
10×RT Random Primer	2 µL	1×
Multiscribe™ Reverse Transcriptase	1 µL	2.5 µg/µL
Nucleasefree water	4.2 µL	
Sample uterine RNA	10 µL	3.2 µg
Total	20 µL	

### Primer design

All the primers for the stem cell markers were selected from a published literature [8]. Before ordering, all the sequences were “BLAST” searched using NCBI database and following five primers were selected OCT4, SOX2, NANOG, KLF4, and GAPDH. This was done to make sure that all the primers are specific to only corresponding canine genes. Primers with corresponding sequences are shown in Table-2. All primers were ordered through MWG Eurofins. The concentration of the stock primers was 100 pmol/µL. The stock solutions were further diluted to make 10 pmol/µL working solution. Melting temperature for each primer was fixed (OCT4 - 53°C, SOX2 - 50°C, NANOG - 55°C, KLF4 - 60°C, and GAPDH - 55°C).

**Table-2:** RTPCR primer sequence.

Name of gene	Sequence of primer (Forward)	Reverse	Amplicon size
OCT4	TCGTGAAGCCGACAAGGAGAAG	AGAACATGTTCTCCAGGTTGCCT	387
SOX2	AACCCCAGATGCACAACTC	CGGGGCCGGTATTTATAATC	162
NANOG	CCTGCATCCTTGCCAATGTC	TCCGGGCTGTCCTGAGTAAG	409
KLF4	CCATGGGCCAAATACCCAC	TGGGGTCAACACCATTCCGT	98
GAPDH	TATCAGTTGTGGATCTGACCTG	ACTCTTCCACCTTCGACGC	172

### Reverse transcription-PCR (RT-PCR)

The cDNA was amplified and cross-checked with the marker (OCT_4_, SOX_2_, and GAPDH) in PCR.

PCR Master Mixes were prepared for all canine experiments using Invitrogen PCR kit. PCR was run in PCR machine (Mastercycler^®^ Pro, Eppendorf) with following PCR reaction mixture (Table-3). An initial denaturation step of 95°C for 5 min followed by 35 cycles of denaturation at 95°C for 1 min, annealing at primer-specific temperatures (OCT_4 -_ 53°C, SOX2 - 50°C, NANOG - 55°C, KLF4 - 60°C, and GAPDH - 55°C) for 1 min, and extension at 72°C for 1 min. The program was completed with a final extension step of 72°C for 10 min. PCR product samples were stored at 4°C until analysis. Negative controls of RNA and water were run alongside to confirm the absence of genomic DNA contamination. PCR products were run on a 2% agarose gel and ethidium bromide. The samples were run through the gel using electrical current (Bio-Rad, UK) to create band separation and visualized using Molecular Imager^®^ Chemi Doc™ XRS+Imaging System, BIO RAD with Image Lab™ software Version 3.0.

**Table-3:** PCR for stem cell specific primer.

Reagent	Sample 1 ° CT4	Sample 2 So×2	Sample 3 GAPDH	Sample 4+ve for GAPDH	−ve for GAPDH
PCR Master Mix	12.5 µL	12.5 µL	12.5 µL	12.5 µL	12.5 µL
cDNA	10 µL cell culture cDNA	10 µL cell culture cDNA	10 µL Cell culture cDNA	10 µL Umbilicus cDNA	
F/R primer	1 µL OCT4	1 µL SOX2	1 µL GAPDH	1 µL GAPDH	1 µL
NFW	1.5 µL	1.5 µL	1.5 µL	1.5 µL	11.5 µL
Total	25 µL	25µL	25 µL	25 µL	25 µL

NFW=Nuclease-free water, PCR=Polymerase chain reaction

### Osteogenesis

The cells were plated at approximately at 1×10^4^ cells per T_25_ polystyrene plate, incubated at 37°C, 5% CO_2_ for 2-4 days to allow growth of adherent cell layer. Dead cells and debris were discarded and the adherent cells washed with 1× PBS. The freshly prepared DMEM 10% media was added and the plate washed at every 2-3 days until the cells achieved 80-90% confluent, and usually, it occurs in 1-2 weeks. Cells were further passaged using 0.25% trypsin-EDTA (Gibco, UK) to loosen cells and analyzed under a microscope for growth. Approximately 1×10^3^ cells/cm^2^ was replaced at each passage in tissue culture flasks. Control cell culture in RPMI 10%+dexamethasone media was grown and passaged in a similar manner. Morphology of cultured cells was studied on 7^th^, 14^th^, and 21^st^ day in both DMEM 10% media and RPMI 10%+dexamethasone. After 3 weeks and 5-6 passages, the cells in DMEM medium were split with trypsinization as follows. Media was pipetted out and discarded. 5 ml of 0.05% trypsin were added to T_25_ polystyrene plate. Shacked and incubated in a CO_2_ incubator for 5 min. T_25_ flask was taken out, pipetted 3-4 times and transferred to a 14 ml Tarson tube, and centrifuged at 1500 rpm for 5 min at room temperature. Then, the supernatant was discarded and pellets shook for 2-3 times.

10 ml of osteogenic differentiation medium containing ascorbic acid 2-phosphate (50 mM), dexamethasone (100 nM), *b*-glycerophosphate (10 mM) (Sigma-Aldrich, USA), and 10% FBS in LG-DMEM was added, shacked, and the cells were then seeded into T_25_ polystyrene plate. Growth and differentiation of cultured cells were studied in control and the differentiation media for at least 3 weeks. The calcium deposition was detected by staining with Alizarin Red-S. Osteogenic differentiation media was discarded from T_25_ flasks. The cells were briefly washed with 1× PBS followed by fixation with ice-cold ethanol (70%, Merck) for 1 h at 4°C. Following Milli Q H_2_O rinsing, the cells were stained for 10 min with 50 mM Alizarin Red-S in water (pH 4.2) at room temperature. Cultures were then rinsed 5 times with Milli Q H_2_O succeeded by a 15 min wash with 1× PBS to reduce non-specific staining following which the stained cells were photographed. Control cells did not stain red. This indicates that endometrial derived cells have multipotent differentiation capacity and self-renewal ability.

### RNA extraction from uterine tissue

**Figure-4:**
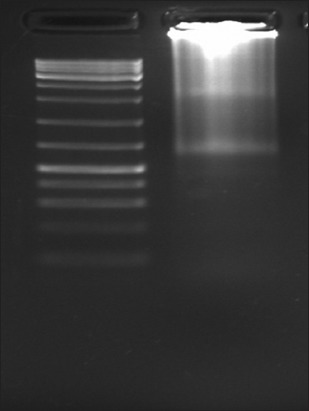
Agarose gel electrophoresis of uterine RNA.

Uterine tissue sections collected during routine ovariohysterectomy procedures in liquid nitrogen, 1% PBS and RNA later were processed in the laboratory for isolation of RNA using TRIzol^®^ extraction protocol involving homogenization, phase separation, and RNA isolation. Finally, the collected RNA pellet was resuspended in 50 µL Riboreserve™ RNA storage solution (Amresco). The eluted RNA was quantified, and quality checked in a spectrophotometer with sample diluted at 1:50. RNA quality was checked through 1.5% Agarose gel electrophoresis by taking 5 µL sample RNA with 1 µL of dye in the well. Band separation was visualized by Molecular Imager using Image Lab™ software version 3.0 (Figure-4). The extracted RNA was reverse transcribed using the protocol outlined in the High-capacity cDNA Reverse Transcription Kit (Applied Biosystem, USA) and prepared cDNA stored at −20°C. Primers for stem cell marker (previously prepared) were used for RT-PCR.

### RT-PCR

The cDNA was amplified and cross-checked with the marker in PCR in a sequential manner. PCR reaction mixtures were prepared for all canine experiments using the PCR kit (Invitrogen, USA) (Table-4).

**Table-4:** PCR reaction mixture with sample from umbilicus.

Reagent in PCR kit	Amount
10 × RT buffer	5 µL
MgCl2	1.5 µL
dNTP	1 µL
GAPDH primer F/R	2 µL
Taq DNA polymerase enzyme	0.5 µL
cDNA from uterus/umbilicus	10µL
DEPCtreated water	30µL
Total	50 µL

PCR=Polymerase chain reaction

The following protocol was found to be optimal for the reactions. An initial denaturation step of 95°C for 5 min was followed by 35 cycles of denaturation at 95°C for 1 min, annealing at primer-specific temperatures for 1 min, and extension at 72°C for 1 min. The program was completed with a final extension step of 72°C for 10 min. The samples were stored at 4°C until analysis and negative controls of RNA and water were run alongside to confirm the absence of genomic DNA contamination.

PCR products were run on a 2% agarose gel and 2% ethidium bromide. The samples were run in electrophoresis tank connected to a power pack (Bio-Rad, UK) to create band separation and analyze with Molecular Imger^®^ Chemi Doc™ XRS+Imaging System.

### RNA extraction from umbilical cord matrix (positive control)

Umbilicus which was collected in liquid nitrogen and kept at −80°C was taken out, triturated with pestle and mortar. RNA extraction was done the following guidelines of TRIzol® extraction protocol for RNA extraction from the tissue sample. Four samples of umbilicus were homogenized (using power homogenizer) separately in 1 ml of TRIzol and processed in the same manner as RNA was extracted from the uterus. This was followed by phase separation, RNA precipitation, washing, and resuspension, or elution with Riboreserve RNA storage solution. 50 µL RNA was prepared from each of four umbilical samples and stored at −80°C. 2 µL of the RNA from each sample was utilized for quantitative estimation in a spectrophotometer (Biophotometer plus, Germany) with 1:50 dilution ratio. The RNA quality was checked through 1.5% agarose gel electrophoresis by taking 5 µL RNA from each sample with 1 µL of dye in each of four wells. Band separation was analyzed for four RNA sample. The procedure for cDNA synthesis was done following High-capacity cDNA Reverse Transcription Kit as described previously and RT-PCR amplification of all the five primers done.

### RT-PCR analysis

The cDNA synthesis was further amplified in PCR system using five primers (OCT4, SOX2, NANOG, KLF4, and GAPDH), and the product was analyzed in Gel Doc Molecular Imager.

## Results

### Tissue sectioning and cryostat picture

Uterine mass which was collected in the liquid nitrogen and stored at −80°C was sectioned in the cryostat and stained with H and E following the protocol of H and E staining protocol of frozen section. Photographs were taken before and after scrapping to evaluate the portion of the uterus used for cell culture, and it was seen that the innermost endometrium layer was scrapped and utilized in the cell culture development.

### Primary culture of canine endometrium-derived MSCs

**Figure-5:**
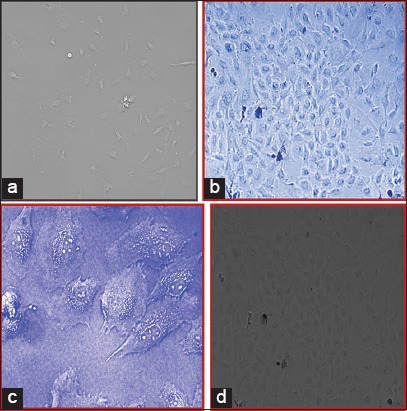
(a) Photomicrograph of cells in Dulbecco’s modified Eagle’s medium (DMEM) on 7^th^ day, 10×. (b) Photomicrograph of cells in DMEM on 14^th^ day, 10×. (c) Photomicrograph of cells in DMEM on 14^th^ day, 40×. (d) Photomicrograph of cells in DMEM on 21^st^ day 10×.

Canine uterus which was collected during routine ovariohysterectomy procedure in 1× PBS was used for the development of primary cell culture on the same day. The cells were seeded in a new flask when they reach 80-90% confluency. The medium was changed weekly twice during which the dead cells and debris get discarded. Non-adherent cells (non-mesenchymal) floated freely and were washed out along with the debris. Photomicrographs were taken at regular intervals on 7^th^, 14^th^, and 21^st^ (Figures-5a-d and 6a-c) days to have a clear outline of changing the shape of the monolayer at the weekly interval and prolificacy of the cell lines.

**Figure-6:**
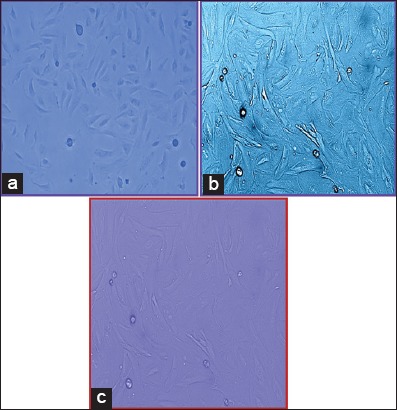
(a) Photomicrograph of cells in RPMI+dexamethasone on 7^th^ day, 10×. (b) Photomicrograph of cells in RPMI+dexamethasone on 14^th^ day, 10×. (c) Photomicrograph of cells in RPMI+dexamethasone on 21^st^ day, 10×, and on 14^th^ day, 10×.

Some of the cells also developed colonies indicating colony-forming unit which is the characteristic of mesenchymal stem cells. The canine endometrial-derived cells displayed spindle-shaped morphology which is a characteristic of MSCs and was adherent to the plastic culture surface (plasticity). MSCs adhered naturally to tissue culture plastic over 2-4 days. It was observed that canine MSCs typically senescence between passage 4 and 6, at which point the cells increased in size and the spindle projections would fatten indicating their maturity. Of two different culture medium, it was seen that cells grown in medium supplemented with dexamethasone showed a number of colonies than DMEM medium.

### Osteogenesis

**Figure-7:**
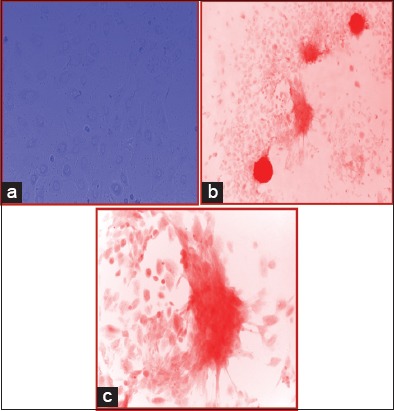
(a) Photomicrograph of cells in osteogenic differentiation media 7^th^ day (10×). (b) Photomicrograph of cells grown in ODM stained with Alizarin Red-S staining (10×). (c) Photomicrograph of cells grown in ODM stained with Alizarin Red-S staining (40×).

**Figure-8:**
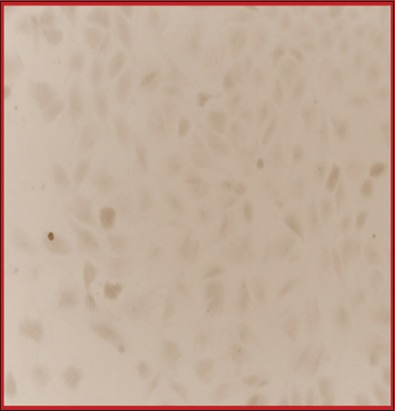
Photomicrograph of cells grown in ODM stained negatively with Alizarin Red-S staining (10×).

T_25_ culture flask having basal culture medium as DMEM+FBS 10% grown for 3 weeks was changed to osteogenic differentiation media, and the cells were cultured for at least 3 more weeks in osteogenic differentiation media. While growing in the induction media, the cells show some morphological differentiating feature and cells also proliferate in this induction media. There was thrice change in induction media before being stained for calcium crystal deposits. Alizarin Red-S staining, which positively stains calcium depositions, was used to detect differentiation. The basal culture medium containing DMEM+FBS 10% was used as a control condition. Under differentiation conditions, there was strong, positive Alizarin Red-S staining (Figure-7a-c). However, all the colonies present on the same disc were not positively stained, but negative staining was observed under control condition (Figure-8).

### Proliferation ability

Cells in the T_25_ culture flask having RPMI 1640+10% FBS+dexamethasone which was maintained in the same culture condition showed a decrease in the proliferation capacity as the cells were passaged from 3 to 10.

### RNA extraction from cell monolayer

The cells grown in 30 mm^2^ Petri dish containing basal culture medium RPMI 1640+10% FBS+dexamethasone was selected for RNA extraction on the 14^th^ day. The RNA was extracted from the monolayer cells following the illustra™ RNAspin Mini RNA Isolation Kit (GE healthcare) guidelines. A 50 µL of RNA was prepared and stored at −80°C for further use. 2 µL of isolated RNA was utilized for quantification using a spectrophotometer and the reading was 1.42 indicating the average quality of RNA. 5 µL of freshly prepared uterine RNA was run on a 1.5% agarose gel to check RNA band and quality. Band separation was analyzed with UV transilluminator and then visualized using Molecular Imager^®^. A faint RNA band was observed in the gel.

### cDNA synthesis and RT-PCR analysis

**Figure-9:**
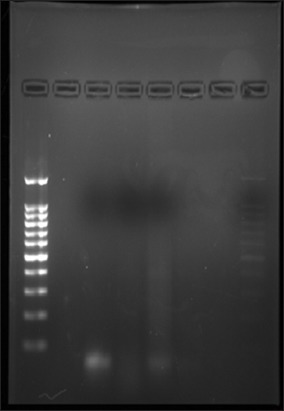
Agarose gel electrophoresis of OCT4 in culture cell, negative for SOX2.

A 10 µL of endometrium RNA was utilized for preparation of cDNA reaction mixture which then processed in the thermal cycler to form 20 µL of cDNA following the protocol of High-capacity cDNA Reverse Transcription Kit. Then, 20 µL cDNA was diluted with 80 µL of DEPC treated water to prepare 100 µL of cDNA with a final concentration of 500 ng/µL. PCR was run in PCR machine with 10 μL of sample cDNA in a PCR reaction tube (Axygen, USA). At first, GAPDH was amplified at 55°C with the umbilicus as a positive control. Band separation was observed in Molecular Imager^®^. Bands appear for GAPDH at 172 bp. Then, PCR was done for other two Primers (OCT4, SOX2) (Figure-9). In this, SOX2 expresses negatively.

### cDNA synthesis and RT-PCR analysis of stem cell primer of umbilical RNA

50 µL of umbilical RNA prepared from four samples were centrifuged and the supernatant discarded. Pellet was again eluted to 50 µL of RNA by mixing with Riboreserve™ RNA storage solution. The cDNA was prepared following High-capacity cDNA Reverse Transcription Kit (Applied Biosystem, USA) protocol. Then, cDNA was amplified by PCR machine.

**Figure-10:**
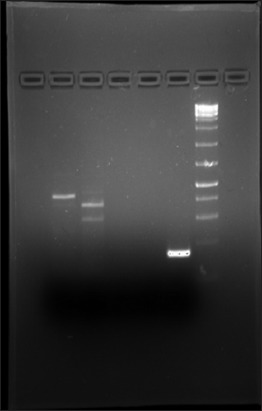
Agarose gel electrophoresis GAPDH, OCT4, SOX2, KLF4, and NANOG of umbilicus RNA.

PCR was run first with GAPDH (55°C) only which results in a positive expression pattern as distinct bands were seen in agarose gel. Then, primer amplification was done for OCT4, SOX2, NANOG, and KLF4 with annealing temperature set at 53°C, 50°C, 55°C, and 60°C, respectively. Positive expression pattern was observed for OCT4 (387 bp), SOX2, and GAPDH (172 bp). The NANOG and KLF4 express negatively as no bands were seen in their respective well (Figure-10).

## Discussion

The canine uterus consists of perimetrium, myometrium, and endometrium. The perimetrium is the outermost serosal or connective tissue layer of the uterus which holds it in position within the pelvic cavity. The myometrium is the muscular layer supplying blood and lymph to all other layers. The particular importance of the study was the innermost endometrium consisting of the superficial functional zone and thin deep basal zone. The superficial zones degenerate partially or fully in each reproductive/estrus cycle, whereas the basal zone persists throughout the cycle and replaces the upper layer when lost. Thus, the canine endometrium can be defined as a cyclically regenerating mucosal tissue comprising of glands and extensive vascularized stroma.

Studies by Gargett *et al*. [17] and other scientist have shown that both human and murine endometrium may contain adult stem cells such as clonogenic cells [18], side population cells [19,20], and some cells that can differentiate into osteocytes, adipocytes, and chondrocytes [21] in human endometrium. Gargett *et al*. [17] showed in their study about the presence of epithelial progenitor cells having MSC like properties.

The fact that human endometrium contains stem cells in the basal layer and stroma while canine endometrium contains cells in the basal zone which restores cells of the superficial zone at the time of need which encourages us to hypothesize that the “basal layer of canine endometrium contains cells which may have the characteristic of stem cell-like properties.”

Various researchers and workers have isolated stem cells from diversified organ starting from blastocyst of embryo (for ESCs), umbilical blood to adult tissue such as bone marrow, adipose tissue, liver, heart, skin, intestine, lungs, deciduous teeth, and also birth associated tissue such as umbilical cord blood and placenta (amnion, chorion, and allantois) (for MSCs). However, the amount of stem cells that have been isolated from these tissues is not promising. Furthermore, canine stem cells have been studied for use in cell therapy in few cases [9,22,23] although the amniotic membrane has clinical applications in covering wounds, burn lesions, and ocular surface reconstructions [24]. Therefore, a constant and established source of stem cells is the need of the day which can only be achieved through collection, separation, purification, and proliferation of cells derived from endometrium obtained anytime through routine ovariohysterectomy procedures carried out in the Animal Birth Control Program.

In the present study, the photographs were recorded during cryostat section of three layers of uterus through H and E straining, and then after scrapping, the endometrium through H and E staining (only perimetrium and myometrium were seen) gives the information that the scrapped cells cultured were actually from the basal and superficial layer of endometrium and not from other two layers.

Further, the primary cell culture from these scrapped cells was developed and maintained up to at least ten passages in the basal medium. The cells were isolated and cultured from three out of four different uterine samples (the rate of success of propagation was 75%). All the isolated cells showed very similar cell morphology and ability to be subcultured. Although the initial cell culture consisted of both fibroblastoid and non-fibroblastoid cell types, only the fibroblastoid population remained after enzymatic digestion and passage. Therefore, the endometrium-derived stem cells can be successfully isolated and expanded *in vitro*.

During maintenance and passaging of cells, it was learned that these cells adhered to the cell culture flask in 2-3 days and growth takes place exponentially indicating prolificacy characteristic (one of the functional properties) of mesenchymal stem cells. Flasks which were subjected to osteogenic differentiation media other than basal culture condition showed a good level of induction to osteogenesis. To confirm the differentiation ability in osteogenic medium, the flask containing cell monolayer was stained with Alizarin Red-S (pH 4.2) and showed strong positive reaction, indicating differentiation into one or more lineage (another functional property of stem cells in culture condition).

With the demonstration of functional properties *in vitro*, the properties of these cells have been further evaluated by isolating RNA from one of the Petri dish through the guided protocol and subsequently synthesizing the cDNA and RT-PCR analysis for amplification of five publicized primer (OCT4, SOX2, NANOG, KLF4, and GAPDH). It adds to our evaluation that OCT4, SOX2, and GAPDH which were considered to be primary embryonic stem cell markers and expressed by almost all adult stem cells of the body were also expressed by these cell types. NANOG and KLF4 were found negatively expressed as for other MSCs. This suggests that the endometrium-derived stem cells are very primitive cells. If this belief is correct, then the renewal and differentiation capacity of endometrium stem cells are more extensive than ASCs. Our morphology and differentiation study indicated the presence of stem cells in uterine cells harvested from the fresh canine uterus; however, we failed to detect any stem cell marker through normal PCR method and this might be due to the following reasons. (a) In the collected uterine endometrium, the number of stem cells might be very less compared to the umbilicus. (b) The technique might not be very sensitive to detect low abundance cells. (c) In the cultured cells at the time of RNA isolation, many cells might have already differentiated. This is also supported by the fact that our Alizarin Red-S staining failed to stain all the cells in the culture dish.

In this work, the umbilicus was taken as a positive control for expression of these stem cell marker based on the report of Lee *et al*. [6] and compared with endometrial-derived RNA, PCR product, and amplified primer with its corresponding counterpart.

## Conclusion

These results showed that the isolation of stem cells from canine endometrium expresses the characteristic of MSCs and self-renewal ability. The presence of stem cells in the endometrium could have tremendous implications. Although this is a basic and preliminary study of its types, the initial data on the differentiation capabilities of endometrium derived stem cells are promising and there may be important therapeutic uses for these cells. Along with this, the ease of accessibility, lack of ethical concerns, and abundant availability of canine-En-MSCs, it shows an attractive and alternative source of adult stem cells for further research and clinical applications.

## Authors’ Contributions

AKS prepared the study design and carried out research under the supervision of JKD and SN. The manuscript was drafted by AKS and revised by JKD. All authors read and approved the final manuscript.
